# The Nature of and Choice of Methods in the Treatment of Uterine Cancer

**Published:** 1893-06

**Authors:** Z. H. Evans

**Affiliations:** Traverse City, Mich.


### THE NATURE OF AND CHOICE OF METHODS IN THE TREATMENT OF UTERINE CANCER.

By Z. H. EVANS, M. D., Traverse City, Mich.

CANCER is like a circle, whose circumference is everywhere and its center nowhere. Cancer thus defined fully explains why our efforts to disintegrate a part of ourselves from ourselves should result in constituting the great opprobrium of surgery, as well as leaving a disease in the world with the proportions of a pestilence. The great difficulty encountered at perfect eradication of cancer is, and always has been, the lacking the idea of the remedy to be inserted into the idea of the disease. Therefore, a knowledge of the etiology of cancer is of paramount importance, and all other questions regarding it are dwarfed into insignificance. The period in which the genius forges the key, and with it unlocks the mystery of cancer, will ever stand alone in its record as accomplishing the greatest good to the greatest numbers, and well can we afford to give up the discoveries of a century for the dazzling picture revealed.

The local elements of cancer have taught us nothing. We must first comprehend the law tabernacled within the elements before we can even hope to explain the origin of cancer. The pathologist has, perhaps, gone a little deeper into pathological changes with his lenses, and the chemist has gone further into matters with his retorts than ever has been accomplished before, but it is a fact, nevertheless, that the cause of cancer remains today as great a mystery as it was in the days of Galen.

The power that creates and authors cell-life has never been placed on the dissecting-table, and touched by the surgeon's knife, nor carried to the laboratory, and there made to yield its pedigree to the genius of human investigators. Finite power becomes weak and lost in the dark recesses of Nature when we try to penetrate into the unrevealed methods of the workmanship of a hand that is divine. The mysteries of the sidereal world fade into oblivion, when contrasted with that mysterious motor-power that causes cell-life to go into rebellion and become active participants in the performance of a function that is so destructive to human life. Cancer-cells, like the seeds of plants, have a period of development, growth, and decay, but, unfortunately, though they perish they leave behind germs pregnated, and thus they are rendered capable of perpetuating their species, and herein is anchored the difficulty of its successful therapeutical management. The question now before us, naked and deprived of all collateral and advantageous issues, is, What is cancer? Is it in its origin simply a local disease and the constitutional disturbances merely affected? Or, is the starting point in the constitution and the local lesion its results? This is a question of the greatest interest and importance, and, in my judgment, admits of but one distinct reply.

The greatest evil, or the one to be most dreaded, is the huge bulk of the ill-digested, pathological theories that have been put forward from time to time, and by their respective sires taught to explain the origin of cancer. Thus far, unfortunately, all of these theories have utterly failed to stand the test of practical utility in the broad untrammeled arena of clinical experience. These theories are not, however, barren of results, for they have done much towards joining rashness to ignorance. A combination thus effected at once becomes more dangerous to suffering humanity than the diseases from which humanity is suffering. Professor Roswell Park, of Buffalo, N. Y., has, in the New York *Medical Journal* for March 4th, given us a very complete and exhaustive *resumé* of the theory of parasitic cancer; and, at the conclusion of his paper, he says: "You may better appreciate when you recall that my home is in Western New York, in a limited area, where the death-rate from cancer is greater than in any other part of our continent." Dr. Park's predecessor, the late learned Professor Julius F. Miner, with others, shared in the opinion that cancer, like consumption, lupus, leprosy, and scrofula, was nothing more or less than the local manifestations of some one of the many by-products of degenerated syphilis. Therefore, if syphilis is the cause of cancer, then Prof. Park is not only sustained in his statement that the death-rate from cancer is greater in Western New York than in any other part of our continent, but it degrades the Queen City of Buffalo, by crowning her the metropolis of syphilis.

The origin of cancer, according to Cohnheim, that there is but one way in which it is conceivable that a new growth should arise in the adult, and that is by a portion of embryonic tissue having become arrested in its development during fetal life, and having remained shut off until its dormant capacities have ultimately aroused into activity. Such tom-foolery as this no surgeon can comprehend until he first dispossesses himself of the idea that he is an intellectual being, and to accept such a dogma his mind at once becomes entombed in as great a mystery as the idea that the first man was made in an instant from dust, or the greater Adam was begotten without the aid of an earthly father. These theories that are born and brought into the world by the self-constituted, romancing pathologist, oftentimes appear quite rich in original research, until finally some day clinical experience proves the falsity of the doctrine and then the theory becomes a mere phantom, and its author is converted into a pathological ass, and as such he becomes a show before the surgical world.

Writers upon the subject of cancer can do nothing but to offer material for thought, and if they are honest and fully conversant with its clinical history, they are free to admit that they are discoursing upon a subject of which they have not learning. While it now seems no longer doubted (by a few, at least,) a cancer may become a purely local disease, after the diathesis that first produced the local lesion becomes permanently exhausted, and then the local lesion can assume the role of a benign tumor, deriving its nutrition from the blood previously restored to health in the same manner as other growths receive their nutrition. (I have seen one cancerous tumor thus transformed.) Upon the other hand we oftentimes see what is called a benign tumor grow out of its original condition and take on another. As it rises through and above the condition in which it first existed, it then seems to fall away and dissolve and becomes transformed or modified, and now other elements are added, until sometimes suddenly, sometimes gradually, until finally we see the growth entirely in a new set of circumstances. The original tumor thus transformed is made up partly of the old, but the old so altered as to be almost unrecognizable. At this stage of its development it is not slow in giving warning, by appropriate signals, that it is ready for the struggle, and it commences by first reaching out in all directions and confiscating all the issues in its immediate neighborhood, and converting them to its own aim, which is, broadly speaking, death.

I am, however, clearly of the opinion that all of these so-called benign tumors that assume a malignant character have had, from their very earliest development, cancerous material tabernacled within their anatomical make-up, only existing to a lesser degree of malignancy. Surgery says that scirrhus is rarely, if ever, encountered in childhood, and never seen in infancy, but it does appear in some pedigrees after the fourth year of life has passed, bordering almost, if not quite, on an epidemic, and how prone it is to attack the uterus and breast as these organs are passing into that condition known as senile repose, is well-known to every surgeon of clinical experience. Therefore, if youth is the only barrier against cancer, our best hope for the future lies, without doubt, in the complete rejuvenation of the cancerous race; and for the accomplishment of this purpose Brown-Sequard has given us the remedy which he calls the *elixir of life* in the substance of squeezed testicle juice. But, though unfortunately for the cancerous race, the only effect the remedy has had when used upon this side of the Atlantic, at least, has been to create abscesses and make *mouthpieces* out of the few who have been foolish enough to resort to its use.

It matters not by what method the local lesion of cancer is destroyed, nor does it matter how rapidly the local wound may have healed, and however flattering may be the temporary restoration of health and strength, the annals of surgery prove that the poor suffering victims sooner or later pay the debt for the sins of their fathers, in death. Granting, for the sake of argument, that cancer is, at first, only a local disease, and only becomes constitutional in its more advanced forms, we now arrive at a point in the discussion of the subject wherein we find ourselves with some of the difficult and perplexing problems that a surgeon is ever called upon to consider, viz., to state at what stage of the disease cancer ceases to become local and assume its constitutional character. There is no such thing as dodging this question. There must necessarily exist a border-line between all normal and abnormal tissue, as there is a border-line between the animal and vegetable kingdom. Schröder once stated his ability of distinguishing, by the touch, with perfect certainty, very small infiltrations, such as occur principally along the lymphatic vessels. Schröder's own operative results clearly prove that, when he made that statement, he was laboring under as great a delusion as the operation of total extirpation of the uterus, for cancer is *barren of permanent results*.

Another difficulty standing in the way of operating early, before the disease becomes a general one, and that is to be able to diagnosticate malignant from non-malignant affections of the uterus. This is a matter not only requiring the eye of an eagle, but he must be a man endowed by Nature with the assumed touch of a Schröder, as well as possessing the real brains of a Paget, in order to early detect the pathological changes peculiar to each. I admit that the removal of a cancerous uterus is an operative feat peculiar only to the century that is now fast drawing to a close. Neither can it be denied, when I say, that I voice the sentiments of a few at least who say that any surgeon, and it matters not to me who he may be that resorts to the operation of total extirpation of the uterus with the view of curing cancer, where there exists even to a slight degree cancerous material infiltrated beyond the boundaries of the uterus, shortens the life of his victim, and by so doing he becomes a murderer—nay, even worse, for he, by his operation, adds torture to murder. Therefore, I say, in the absence of any known cure for any given disease, it becomes our duty to suffering humanity to select a method of treatment that experience has proven will secure to the patient the greatest number of days, with least possible amount of suffering, and those who do this best serve the interest of those who place their lives, as well as their confi[illegible]ence, in our keeping.

We have, today, three methods of treatment for uterine cancer. First, amputation or excision of the diseased structures by the knife or scissors. Second, actual or chemical cauterization. Third, extirpation of the entire uterus. In comparing these three methods of treatment now in vogue, I shall have nothing to say as to the primary mortality. It is the remote results that most concern us at this time. Dr. W. H. Baker, of Boston, has reported the final results of twelve cases of high amputation for cancer of the uterus. In one case the disease returned in two months ; in another, the disease returned almost immediately, while in a third, the patient died in three months after the operation, from cancer. In a fourth, the disease returned in nine months. Six years later, from this report, Dr. Baker states that five of these women were well and free from cancer. Dr. T. A. Reamy has given us his results of fifty-five cases. In fourteen amputations there was no recurrence of the disease within one year ; in eight, there was recurrence in two years ; in four, there was recurrence at periods varying from two to four years ; in twenty, there was no recurrence from two to eight years.

Hofmeier has published a table of ninety-six cases in which partial amputation was done at Berlin. Nineteen of these cases were living at the end of four years after operation. Pawlick has reported 136 cases of cancer of the cervix, treated at Vienna, by means of the galvano-cautery. Thirty-three of these women were well and free from the disease, at periods varying from two to twenty-one years. Dr. John Byrne, Brooklyn, has reported the final results of 367 cases of cancer of the uterus treated by the galvano-cautery; in thirty-six cases the average periods of freedom from return was eight years and seven months. Thirty remained well over five years. Of the entire number, 367, over one-half remained free from three to eight years.

*Hysterectomy.*—Fritsch has given us the final results of sixty cases of total extirpation for cancer, and he has been honest enough to tell us that, at the end of three years, he had only two patients alive. Ferrier has given us the results of eighteen operations of total extirpation of the uterus for cancer, and at the end of *two years* only *four* patients were alive. Prof. August Martin, Berlin, has published to the world the results of 214 cases of total extirpation of the uterus for cancer. Of these, Leopold had forty-two; Schröder, forty-six; Fritsch, sixty; and Martin, sixty-six. Out of the entire number, *only five were alive at the end of four years.* Doctor Mary Ann Dixon Jones, Brooklyn, in the *American Journal of Obstetrics* for April, 1893, making use of Martin's report, as quoted above, to appear as though the results obtained favored the operation of total extirpation of the uterus for cancer, says: "Leopold, Schröder, Fritsch, and Martin, out of 214 operations of total extirpation of the uterus for cancer, had, at the end of one year, thirty-five patients living; at the end of two years, twenty-five; and, at the end of three years, twenty." I am a little curious to know why it is that Dr. Jones should only give us the result of these cases for three years, when she could, just as well as not, have given us the report for the fourth year, unless it be that she was afraid that the final report would throw discredit upon the operation so great as to deform the annals of surgery beyond repair, which it does. Again, she says: "Martin, in his late work, out of sixty-six cases" (same number as above quoted), "eleven died under the influence of the operation, thirty-one remained free from recurrence of the disease. This gives, therefore, as a result, seventy per cent. of cures."

Comment is not necessary, for we are told "by their fruits ye shall know them." Again she says: "Langenbeck's historical case —one of the first vaginal hysterectomies performed—lived free from the disease for twenty-six years after." For the benefit of science and the enlightenment of Dr. Mary Ann Dixon Jones, I will quote briefly from the report of this historical case as given by that scholar, Prof. T. Hawkins-Tanner. He says: "Extirpation of the uterus has been practised on some twenty-six occasions for the cure of cancer, but I am only acquainted with one well authenticated report of its having been really successful, though in four instances the patients recovered from the operation. In the successful case the woman remained well for twenty-five years (not twenty-six)."

*But it must be remembered that there was a previous procidentia of the organ*, so that the operator, Conrad J. M. Langenbeck, had a comparatively easy task; while, without being hypercritical, *a doubt may be suggested as to the correctness of the diagnosis*. The details of the case given by Professor Max. Langenbeck, in his thésis Ditotins Utero-Extirpation, Göttingen, 1842: "One successful result, however, from a very dangerous operation cannot outweigh a number of failures; failures, be it remembered, implying death within forty-eight hours in fifteen out of twenty-two fatal cases. Hence, it is almost unnecessary to say that *no* practitioner in the present day would be justified in following the example of the elder Professor Langenbeck, Recamier, and Blundell, with regard to this operation."

This protest was written some twenty-five years ago, but it applies today with the same and equal force as it did when it was written. John Williams, London, in 1890, after a thorough study of all attainable statistics, put the average of *permanent cures* at twenty-eight per cent. Schante, Prague, in 1890, estimates forty-seven and one-third per cent. of permanent cures of all cases operated upon for cancer of the uterus and counted after three years. Pozzi, Paris, in 1890, puts the average of permanent cures from forty to fifty per cent. of all cases operated upon.

Any man, or any class of men, who make the statement that they *cure* from twenty-eight to seventy per cent. of all cases of uterine cancer operated upon, is either suffering from general paresis, or they have fallen victims to that incurable disease known as obliquity of intellectual vision. No radical operation should ever be undertaken which does not hold out a prospect at least as good as that *which can* be claimed for palliation. Is this prospect offered by complete extirpation of the uterus for cancer? I say assuredly not, for the operation is extremely dangerous to life and gives *no* hope of permanent relief, and, therefore, should have no place in surgery.

